# Reduced Subthreshold Characteristics and Flicker Noise of an AlGaAs/InGaAs PHEMT Using Liquid Phase Deposited TiO_2_ as a Gate Dielectric

**DOI:** 10.3390/ma9110861

**Published:** 2016-10-25

**Authors:** Kai-Yuen Lam, Jung-Sheng Huang, Yong-Jie Zou, Kuan-Wei Lee, Yeong-Her Wang

**Affiliations:** 1 Department of Electronic Engineering, I-Shou University, Kaohsiung 840, Taiwan; kylam@isu.edu.tw (K.-Y.L.); jshuang@isu.edu.tw (J.-S.H.); gp30083@msn.com (Y.-J.Z.); 2 Department of Electrical Engineering, Institute of Microelectronics, National Cheng-Kung University, Tainan 701, Taiwan; yhw@ee.ncku.edu.tw

**Keywords:** AlGaAs, pseudomorphic high-electron-mobility transistor (PHEMT), TiO_2_, flicker noise

## Abstract

This study presents the fabrication and improved properties of an AlGaAs/InGaAs metal-oxide-semiconductor pseudomorphic high-electron-mobility transistor (MOS-PHEMT) using liquid phase deposited titanium dioxide (LPD-TiO_2_) as a gate dielectric. Sulfur pretreatment and postoxidation rapid thermal annealing (RTA) were consecutively employed before and after the gate dielectric was deposited to fill dangling bonds and therefore release interface trapped charges. Compared with a benchmark PHEMT, the AlGaAs/InGaAs MOS-PHEMT using LPD-TiO_2_ exhibited larger gate bias operation, higher breakdown voltage, suppressed subthreshold characteristics, and reduced flicker noise. As a result, the device with proposed process and using LPD-TiO_2_ as a gate dielectric is promising for high-speed applications that demand little noise at low frequencies.

## 1. Introduction

The performances of GaAs-based pseudomorphic high-electron-mobility transistors (PHEMTs) have drastically been improved, and have already been extensively used in both low-noise and high-power applications at microwave and millimeter-wave frequencies [1,2,3,4]. However, microwave device manufacturing is still being challenged to achieve high uniformity, high yield, and reliable stability. Hartnagel et al. [5] and Huang et al. [6] describe that major noises in PHEMTs contain thermal noise, shot noise, hot-electron noise, and generation-recombination noise. Shot noise related to the Schottky barrier affects the gate leakage current, and plays an important role in low-noise applications. Hot-electron noise, caused by energetic random electron motion, is associated with impact ionization. Electrons gain energy from supplied electric field and can be randomized by optical phonons, intervalley scattering. If electrons gain enough energy, they can collide with electron–hole pair or impurity and start ionization process. Impact ionization creates current fluctuations and thus is one of the strongest electronic noise sources. Generation-recombination noise induced by the surface recombination centers or defects at the gate terminal/Schottky layer interface can increase the ideality factor, and also produce traps that can contribute to flicker noise. Thus, the Schottky-gate PHEMTs have limited gate leakage current and noise performance levels.

High-κ materials are widely employed as insulators growing on semiconductor to fabricate metal-oxide-semiconductor (MOS) gates for larger gate swing voltages and lower leakage currents [7,8,9]. Titanium dioxide (TiO_2_) is one of the commonly applied high-κ insulators in the semiconductor industry. Numerous methods have been used to successfully deposit TiO_2_ films, such as low-pressure chemical vapor deposition (LPCVD) [10], plasma-enhanced chemical vapor deposition (PECVD) [11], sputtering [12], electron beam evaporation [13], sol-gel deposition [14], and liquid phase deposition (LPD) [15]. Among those methods, LPD is favorable for its low-cost and low-temperature process. LPD-TiO_2_ films have respectively been demonstrated on InP [16], polysilicon [17], glass [18], GaN [19], and AlGaAs [20]. We also previously conducted a preliminary study of LPD-TiO_2_ on AlGaAs without pretreatment [20], and made the LPD-TiO_2_ more compact through sulfide pretreatment [21] and postoxidation rapid thermal annealing (RTA) [22]. However, the low-frequency noise and microwave characteristics of AlGaAs/InGaAs MOS-PHEMT prepared with both ammonium-sulfide-pretreated AlGaAs and postoxidation RTA have not been investigated yet. In this study, an AlGaAs/InGaAs MOS-PHEMT using LPD-TiO_2_ as a gate dielectric after sulfide pretreatment and postoxidation RTA was fabricated, and dc characteristics and microwave performance were discussed.

## 2. Experimental

The proposed device structures were grown through metal-organic chemical vapor deposition on a semi-insulating GaAs substrate. The buffer layer consisted of a 100 nm layer of *i*-GaAs, followed by a 250 nm layer of *i*-Al_0.2_Ga_0.8_As, and a 60 nm layer of GaAs. A 10 nm layer of Al_0.2_Ga_0.8_As with a Si doping density of 4.5 × 10^17^ cm^−3^ and a 2 nm *i*-Al_0.2_Ga_0.8_As spacer layer were then grown on the buffer layer, followed by a 14 nm *i*-In_0.15_Ga_0.85_As channel layer, a 2 nm *i*-Al_0.2_Ga_0.8_As spacer layer, a 18 nm Al_0.2_Ga_0.8_As donor layer with a Si doping density of 1.2 × 10^18^ cm^–3^, a 70 nm Al_0.2_Ga_0.8_As Schottky layer with a Si doping density of 1 × 10^17^ cm^−3^, and a 60 nm GaAs cap layer with a Si doping density of 5 × 10^18^ cm^−3^. Hall measurements showed that the electron mobility was 5900 cm^2^/V·s and the electron sheet density was 2.1 × 10^12^ cm^−2^ at 300 K. 

**Figure 1:**
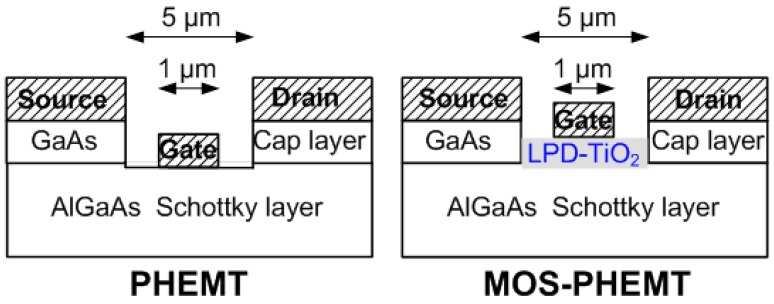
Schematic structures of AlGaAs/InGaAs PHEMT and MOS-PHEMT.

Wafers were first cleaned using acetone, methanol, and H_2_O for 5 min with each solvent. A NH_4_OH:H_2_O_2_:H_2_O (=3:1:50 by volume) solution was used to perform mesa etching, which reached the GaAs buffer layer. Ohmic contacts composed of a 400 nm layer of Au/Ge/Ni alloy (84:12:4 by weight) were deposited through evaporation and then patterned through lift-off processes, followed by RTA at 380 °C for 30 s. After the cap layer and part of the Schottky layer had been etched using the same etchant, the wafer was immediately dipped into a 5% (NH_4_)_2_S_x_ solution for 10 min. For the referenced PHEMT, Au was deposited directly on the sulfide-treated AlGaAs of the Schottky layer. For the MOS-PHEMT, following the (NH_4_)_2_S_x_ pretreatment, the LPD-TiO_2_ was applied on the sulfide-treated Schottky layer at 40 °C [19]. Oxide thickness was approximately 30 nm after postoxidation RTA at 350 °C for 1 min. Finally, the gate electrode was formed through lift-off with Au on the oxide layer. Figure 1 shows the structures of the referenced PHEMT and MOS-PHEMT. The gate length, gate width, and the drain-to-source spacing are 1 μm, 100 μm, and 5 μm, respectively. In addition, the oxide passivated the etched isolated surface wall simultaneously. Microwave on-wafer measurements were conducted from 0.45 to 50 GHz in a common-source configuration by using an Agilent E8364A PNA network analyzer at 300 K.

## 3. Results and Discussion

**Figure 2:**
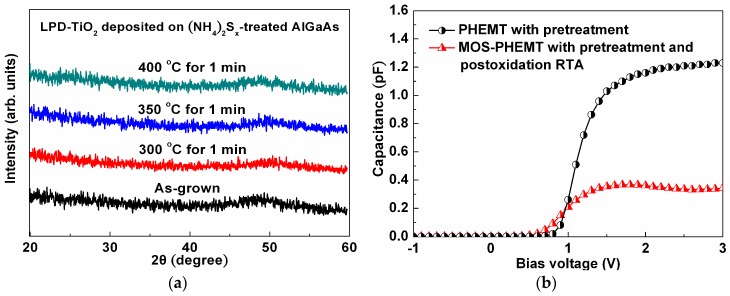
(**a**) XRD spectra of LPD-TiO_2_ deposited on sulfide-pretreated AlGaAs with and without postoxidation RTA; (**b**) C-V comparison for sulfide-pretreated PHEMT and MOS-PHEMT with postoxidation RTA.

Figure 2a shows the X-ray diffraction (XRD) patterns of the LPD-TiO_2_ that was deposited on the sulfide-pretreated AlGaAs with and without postoxidation RTA for 1 min. The XRD patterns did not show peaks corresponding to the anatase or rutile phases when the annealing temperature was raised to 400 °C. The results indicate that the LPD-TiO_2_ lacked sufficient energy to form a single phase or a polycrystal phase at temperatures no greater than 400 °C during the annealing process. Figure 2b shows the 1 MHz capacitance-voltage (C-V) characteristics of the referenced PHEMT and the MOS-PHEMT. The capacitance of the MOS-PHEMT was lower than that of the PHEMT, because the LPD-TiO_2_was in series with the PHEMT. The relative dielectric constant ($\varepsilon_{r}$) of the LPD-TiO_2_ can be calculated using the following equation:

(1) $$C_{\mathrm{OX}}=\frac{\varepsilon_{r}\cdot \varepsilon_{0}\cdot A}{t_{OX}}$$

where C_OX_ is the capacitance of the LPD-TiO_2_, $\varepsilon_{0}$ is the permittivity of free space, *A* is the metal plate area, and $t_{OX}$ is the oxide thickness. The calculated $\varepsilon_{r}$ of the LPD-TiO_2_was approximately 21, fitting the range of amorphous TiO_2_, which was comparable to the $\varepsilon_{r}$ value (24.4) for GaN using the same method [19] and to the $\varepsilon_{r}$ value for polysilicon found by other group [17].

**Figure 3:**
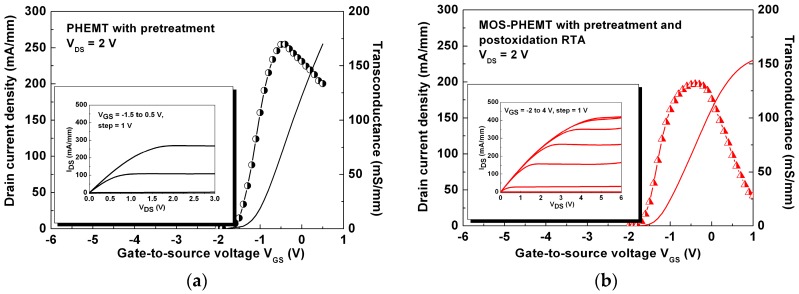
(**a**) Measured I–V characteristics and related transconductance curves for referenced PHEMT; (**b**) Measured I–V characteristics and related transconductance curves for MOS-PHEMT.

Figure 3a,b shows the transconductance (g_m_) and the drain current density (I_D_) as functions of the gate-to-source voltage (V_GS_) at a drain-to-source voltage (V_DS_) = 2 V. The maximum g_m_ values were 170 mS/mm and 132 mS/mm for the referenced PHEMT and the MOS-PHEMT, respectively. However, the gate voltage swing (defined by a 10% reduction of the maximal g_m_) was 0.8 V for the MOS-PHEMT, which was higher than that of referenced case. The insets show the related I_D_-V_DS_ characteristics for both devices. The maximal V_GS_ of the MOS-PHEMT was larger than that of the referenced PHEMT because the MOS-PHEMT had a higher energy barrier between the metal gate and AlGaAs Schottky layer. The maximal I_D_ was approximately 270 mA/mm at V_GS_ = 0.5 V and V_DS_ = 2 V for PHEMT. However, the maximal I_D_ was approximately 200 mA/mm at V_GS_ = 0.5 V and V_DS_ = 2 V, and 420 mA/mm at V_GS_ = 4 V and V_DS_ = 6 V for MOS-PHEMT. The MOS-PHEMT saturation current was less than that of the referenced case at the same V_GS_ because of the voltage drop of the LPD-TiO_2_ underneath the metal gate. However, it was able to induce carriers V_GS_ from 0.5 to 4 V within the channel. By the way, the notable difference of the threshold voltages (V_th_) between capacitor and PHEMT is owing to the different depth of gate recess by wet etchant from different batches.

**Figure 4:**
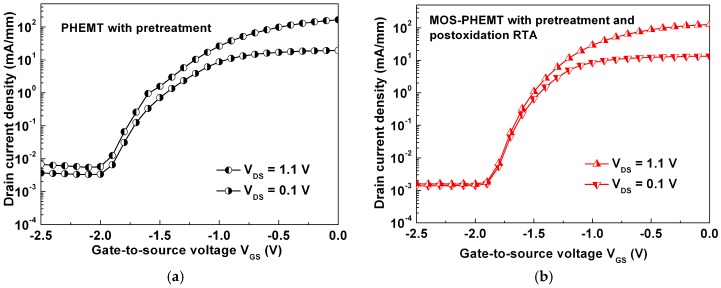
Subthreshold characteristics of: (**a**) referenced PHEMT; and (**b**) MOS-PHEMT with V_DS_ = 0.1 V and 1.1 V.

**Figure 5:**
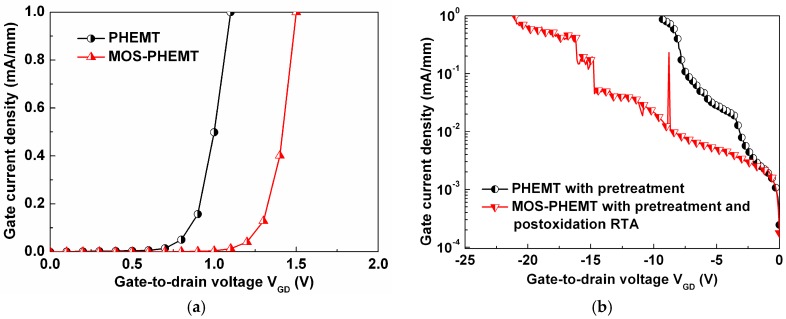
(**a**) Forward gate leakage current density; and (**b**) magnified section of reverse gate leakage current density of typical gate-to-drain diode characteristics for both devices.

The subthreshold characteristics depend on the quality of oxide film and device structure. They determine the ideal off state, and they have effects on power dissipation and IC applications. Figure 4a,b shows the measured subthreshold currents of the referenced PHEMT and MOS-PHEMT, respectively. The subthreshold swing (SS) of the MOS-PHEMT (120 to 125 mV/dec) was lower than that (173 to 194 mV/dec) of the referenced PHEMT. The I_ON_/I_OFF_ ratio of the MOS-PHEMT (8.1 × 10^3^ to 4.1 × 10^4^) was higher than that (4.8 × 10^3^ to 1.5 × 10^4^) of the referenced case, where I_ON_ was I_D_ at V_GS_ = V_th_ + 0.5 V, and I_OFF_ was I_D_ at V_GS_ = V_th_ − 1 V. These results clearly suggest that the MOS-PHEMT suppressed its subthreshold current by reducing the surface recombination current of the LPD-TiO_2_ around the ohmic contact region. That is, the undesirable carrier injection from the source terminal in an off state can be suppressed. Improvements of the SS and I_ON_/I_OFF_ ratio were also associated with suppressed gate leakage characteristics [23], and this association is consistent with the results shown in Figure 5.

The LPD-TiO_2_ caused an improvement in the breakdown voltage associated with the gate leakage current of the typical gate-to-drain diode characteristics, as shown in Figure 5. Figure 5a shows that the turn-on voltage (V_on_) of the MOS-PHEMT, 1.5 V, was obviously higher than that of the referenced PHEMT, 1.1 V. For the MOS-PHEMT, the gate leakage current density was suppressed by approximately two orders of magnitude, and the corresponding reverse gate-to-drain breakdown voltage (BV_GD_) was more than −21.2 V, as shown in Figure 5b. The V_on_ and the BV_GD_ were defined as the voltage at which the gate current reaches 1 mA/mm. Generally, an increased V_on_ accompanies an improved gate voltage swing. The gate leakage current density of the MOS-PHEMT was lower because of the MOS structure and the elimination of the sidewall leakage path passivated by the LPD-TiO_2_.

**Figure 6:**
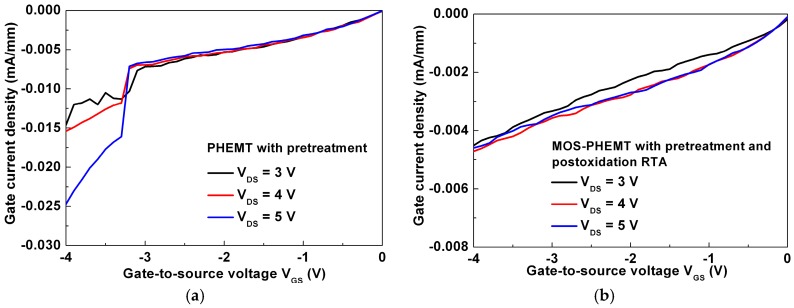
Gate current density versus V_GS_ with different V_DS_ for: (**a**) referenced PHEMT; and (**b**) MOS-PHEMT.

The gate current density, as a function of V_GS_, was measured to obtain insights on the influence of impact ionization. Because of the deep-complex (DX)-center and surface states of AlGaAs, the impact ionization or kink effect is key concern for the AlGaAs/InGaAs PHEMT. A roughly bell-shaped curve is the typical behavior of impact ionization, as shown in Figure 6a for the PHEMT. Marked increases in the gate current clearly occur when devices are biased at higher V_DS_. The gate current densities of the MOS-PHEMT and PHEMT were 4.59 × 10^−3^ mA/mm and 2.47 × 10^−2^ mA/mm at V_DS_ = 5 V and V_GS_ = −4 V, as shown in Figure 6a,b, respectively; therefore the MOS-PHEMT device’s performance was approximately 5.4 times higher than that of the PHEMT. In the referenced PHEMT, significant hot-electron phenomena occurred in the InGaAs channel because of a high electric field near the gate-to-drain region; that is, electrons could obtain higher energy to generate electron–hole pairs through enhanced impact ionizations in the InGaAs channel, which facilitated injection of the holes into the gate terminal [24] or becoming trapped in pre-existing traps. Furthermore, the generation of holes by impact ionization and their further recombination could result in fluctuations of the charges pileup and thus the excess noise. These phenomena also led to increased high-frequency noise at corresponding voltages [25]. In the MOS-PHEMT, the electric field near the gate-to-drain region at the same V_DS_ and V_GS_ improved notably compared with the values of the referenced case, because of the high barrier height of LPD-TiO_2_ underneath the gate terminal. Thus, the improvements of the MOS-PHEMT resulted in a smaller channel electric field and a suppressed impact ionization that further reduced the leakage current density. As mentioned earlier, the suppressed leakage current and impact ionization effect in Figure 6b were expected to improve noise performance.

**Figure 7:**
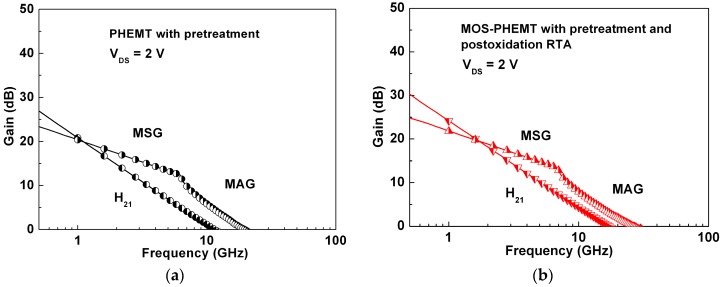
Comparison of microwave characteristics at maximum g_m_ for: (**a**) referenced PHEMT; and (**b**) MOS-PHEMT.

As shown in Figure 7 (different samples from those shown in Figure 3), the measured unity-current-gain cutoff frequency (*f_T_*) and the maximum oscillation frequency (*f_max_*) were 17.3 (11.6) GHz and 26.4 (19.7) GHz at the maximum g_m_ for the MOS-PHEMT (PHEMT). The trend is consistent with the results previously found for E-mode InGaP/InGaAs MOS-PHEMT with liquid phase oxidation (LPO) [26]. The increased microwave performances of the AlGaAs/InGaAs MOS-PHEMT may be attributed to the increase in the ratio of g_m_ to gate-source capacitance (*C*_gs_). Furthermore, the reduction of the surface recombination may also have contributed to the frequency response.

**Figure 8:**
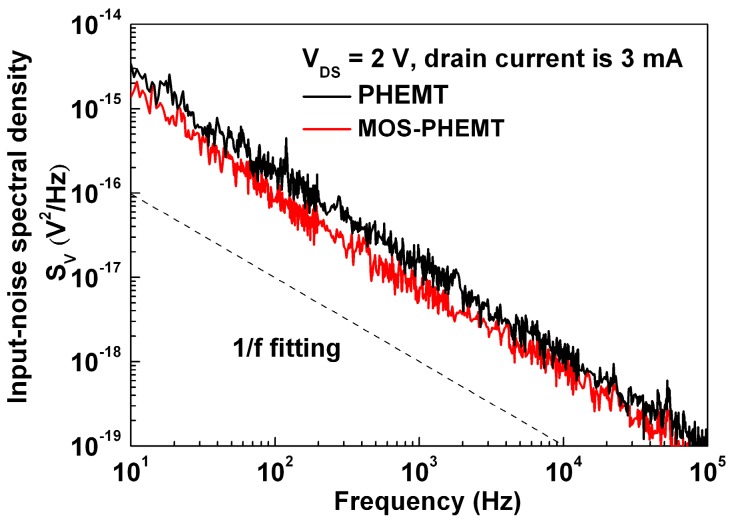
Comparison of low-frequency 1/f characteristics for both devices.

Figure 8 shows the low-frequency flicker noise spectral density (S_V_) characteristics, which were measured using a BTA 9812B noise analyzer and an Agilent 35670A dynamic signal analyzer. On-wafer flicker noise measurements of the referenced AlGaAs/InGaAs PHEMT and AlGaAs/InGaAs MOS-PHEMT were conducted under V_DS_ of 2 V and drain current of 3 mA for frequencies between 10 Hz and 100 kHz. S_V_ can be expressed as follows [27]:

(2) $$S_{V}=\left(\frac{q\cdot \alpha_{H}\cdot v_{sat}}{f^{\gamma }\cdot L_{g}}\right)\cdot \left(\frac{I_{D,\;sat}}{g_{m}^{2}}\right)$$

where *q* is the elementary charge, *α_H_* is the Hooge parameter, *v_sat_* is the effective carrier saturation velocity, *f* is the frequency, γ is the frequency exponent, and $L_{g}$ is the effective gate length. The *α_H_*/S_V_ values at 10 Hz for the referenced case and MOS-PHEMT were 2.8 × 10^−4^/3.4 × 10^−15^ V^2^·Hz^−1^ and 2.7 × 10^−5^/1.4 × 10^−15^ V^2^·Hz^−1^, respectively. The corresponding γ values were calculated to be 1.5 and 1.1, respectively. The higher γ was notably related to generation-recombination noise (i.e., γ = 2). In other words, the LPD-TiO_2_ could passivate dangling bonds to improve the surface state between the LPD-TiO_2_/AlGaAs interfaces, and a reduction of the surface state was observed with negligible low-frequency generation-recombination noise of the AlGaAs/InGaAs MOS-PHEMT.

Table 1 summarizes the dc, low-frequency noise, and microwave characteristics for LPD in this study and previous studies [28,29] and for LPO [30] in AlGaAs/InGaAs MOS-PHEMTs with similar structures but different types of gate oxides. The use of high-K LPD-TiO_2_ with both sulfide pretreatment and postoxidation RTA as a gate oxide and as an effective passivation layer on AlGaAs/InGaAs PHEMT provides new opportunities for low-noise applications.

## 4. Conclusions

This study demonstrates the feasibility of preparing an LPD-TiO_2_ gate with both sulfide pretreatment and postoxidation RTA on AlGaAs/InGaAs MOS-PHEMT near room temperature. Compared with the referenced PHEMT, the MOS-PHEMT had larger gate voltage swing, lower subthreshold characteristics, reduced gate leakage current (with a suppressed impact ionization), enhanced microwave performance, and reduced flicker noise. These features evidence that the proposed device with simple and low-temperature LPD-TiO_2_ gate is suitable for device applications.

## Figures and Tables

**Table 1:** Summary of dc, low-frequency noise, and microwave characteristics of AlGaAs/InGaAs MOS-PHEMTs with similar structures but different types of gate oxides.

Group	This Work	[28]	[29]	[30]
Mode	D-mode	D-mode	D-mode	D-mode
Gate oxide	TiO_2_	SiO_2_	Al_2_O_3_	Oxidized AlGaAs
Oxidation method	LPD	LPD	LPD	LPO
Temperature (°C)	40	40	40	50
Gate length (μm)	1	1	1	1
Maximum V_GS_ (V)	4	4	2.5	4
Maximum I_DS_ (mA/mm)	420	421	433	380
Gate voltage swing (V)	0.8	2.5	2	0.7
SubthresholdSwing (mV/dec)	120–125	125–165	–	–
S_V_ at 10 Hz (V^2^·Hz^−1^)	1.4 × 10^−15^	–	–	–
*f_max_* (GHz)	26.4	–	–	–
